# Volunteers in Ethiopia’s women’s development army are more deprived and distressed than their neighbors: cross-sectional survey data from rural Ethiopia

**DOI:** 10.1186/s12889-018-5159-5

**Published:** 2018-02-14

**Authors:** Kenneth Maes, Svea Closser, Yihenew Tesfaye, Yasmine Gilbert, Roza Abesha

**Affiliations:** 1 Department of Anthropology, Oregon State University, Corvallis, OR USA; 2 Department of Anthropology, Middlebury College, Middlebury, VT USA; 3 Independent Researcher, Bahir Dar, Ethiopia

**Keywords:** Community health workers, Ethiopia, Food insecurity, Water insecurity, Mental health

## Abstract

**Background:**

Many Community Health Workers (CHWs) experience the same socioeconomic and health needs as their neighbors, given that they are by definition part of their communities. Yet very few studies aim to measure and characterize experiences of deprivation, poverty, and wellbeing among community health workers. This study quantitatively examines deprivation and wellbeing in Ethiopia’s Women’s Development Army (WDA), a massive unpaid community health workforce intended to improve population health and modernize the country.

**Methods:**

We conducted a survey of 422 volunteer WDA leaders and community members in rural Amhara state, part of a mixed-methods ethnographic study of the experiences of women in the WDA. The survey asked a variety of questions about respondents’ demographics, education, assets, and access to government services. We also used survey measures to evaluate respondents’ levels of household food and water security, stressful life events, social support, work burden, and psychological distress.

**Results:**

Volunteer WDA leaders and community members alike tend to have very low levels of schooling and household assets, and to be heavily burdened with daily work in several domains. Large proportions are food and water insecure, many are in debt, and many experience stretches of time with no money at all. Our survey also revealed differences between volunteer WDA leaders and other women that warrant attention. Leaders are less likely to be married and more likely to be divorced or separated. Leaders are also more likely to experience some aspects of food insecurity and report greater levels of psychological distress and more stressful life events. They also report slightly less social support than other women.

**Conclusions:**

In rural Amhara, women who seek out and/or are sought and recruited for leader roles in the WDA are a population living in precarity. In several domains, they experience even more hardship than their neighbors. These findings highlight a need for careful attention and further research into processes of volunteer CHW selection, and to determine whether or not volunteering for CHW programs increases socioeconomic and health risks among volunteers. CHW programs in settings of poverty should stop using unpaid labor and seek to create more paid CHW jobs.

## Background

Community health workers (CHWs) are crucial to health systems and are at the center of policies targeting Universal Health Care, Sustainable Development Goals, and the reduction of health inequalities. CHWs by definition are supposed to come from the very populations they serve [1, 2]. As community health workers often target populations living in poverty [3], it follows that CHWs themselves tend to live in poverty and thus experience various forms of deprivation.

Overall, much of the research evidence on community health workers is focused on improving job performance, particularly in a cost-effective manner [4–9]. In this literature on CHW effectiveness, consideration of whether the socioeconomic and health needs facing their communities are directly affecting CHWs themselves remains in the background. While CHWs living in poverty can make a positive impact in their roles [3], their wellbeing or precarity are topics that deserve attention. There is a significant risk that research focused on cost-effective job performance improvements, without foregrounding information on CHWs’ socioeconomic vulnerabilities, can further policies that encourage CHW exploitation and deepen inequality and deprivation [10, 11].

Globally, there is a growing body of qualitative literature that describes the experiences of CHWs, often in social context and often with a nuanced consideration of the gendered dimensions of CHW work [12–17]. But there is limited quantitative work exploring CHW wellbeing and deprivation. To our knowledge, the only studies that have measured food insecurity and/or psychological distress among CHWs have been in Ethiopia. Maes and colleagues reported levels of food insecurity and psychological distress among unpaid CHWs in Addis Ababa [18, 19]. Dynes and colleagues measured food insecurity among Health Extension Workers, Community Health Development Agents, and traditional birth attendants in seven kebeles in Amhara state [20].

Here, we report the results of a survey of food insecurity, water insecurity, workload, and psychosocial wellbeing conducted in 2015 with 422 members and leaders of Ethiopia’s “Women’s Development Army” (WDA) in West Gojjam, Amhara state. It is a measure of how little is known about CHW vulnerabilities that this is the first survey of its kind. We know of no other study that has measured both food and water insecurity among CHWs in Ethiopia or anywhere in the world.

### Ethiopia’s Women’s Development Army

Compared to other nations in Africa and the world, maternal and child mortality rates in Ethiopia were very high at the turn of the twenty-first century. While mortality rates have reduced significantly in the past 15 years, a drop in the maternal mortality ratio (MMR) seen between 2000 and 2005 (from 871 to 673 deaths per 100,000 live births) appeared to stall in 2010 [21]. According to Ethiopia’s Demographic and Health Surveys (DHS), between 2005 and 2011 early neonatal death rates were also stagnant at approximately 37 deaths per 1000 live births [22].

Like many other low-income countries, Ethiopia also faced a critical lack of community level health workers at the turn of the twenty-first century, with fewer than 700 health officers serving a population of over 70 million in 2004 [21]. Ethiopia’s leaders have attempted to address these interlinked problems in part through a set of Community Health Worker-focused investments and reform within the nation’s Health Extension Program. In 2003, the government began to train, deploy and supervise more than 30,000 salaried female Health Extension Workers (HEWs). At the outset of the Health Extension Program in the mid-2000s, the Ethiopian government rhetorically tied the sustainability of primary health care services to the act of creating paid CHW jobs. Dr. Tedros Adhanom, then Minister of Health of Ethiopia and now Director General of the World Health Organization, claimed at the time that the success and sustainability of the Program hinged upon “engaging health extension workers as full-time salaried civil servants” and thereby “moving away from volunteerism” [23]. These statements echoed the WHO’s 2008 recommendation that “essential health services cannot be provided by people working on a voluntary basis if they are to be sustainable” [24]. Ethiopia’s cadre of Health Extension Workers have thus received a salary since the beginning of the program, and they have received modest raises along with other Ethiopian civil servants over the past decade.

Then, beginning around 2011, the government began rolling out an ambitious new Community Health Worker program, aimed to address both the high workload of Health Extension Workers and the fact that, as the government saw it, many families were “lagging behind” in terms of adopting a “healthy lifestyle” [25]. The government announced it would establish what it calls a “Women’s Development Army” (Amharic: *yesetoch lemat serawit*), sometimes referred to (in English) as the Health Development Army or Health Transformation Army.

According to plans, the Army will ultimately incorporate the vast majority (up to 90%) of the adult women living in Ethiopia’s countryside. One woman out of every five households is to become a “1-to-5” (Amharic: *and le ammist*) Women’s Development Army leader, chosen for her status as a “model woman,” a distinction that hinges on having adopted a certain lifestyle deemed healthy and development-minded by leaders in Ethiopia’s central government. A group of approximately five 1-to-5 leaders (hereafter, 1–5 leaders) is in turn led by a “1-to-30 leader” (hereafter 1–30 leader). 1–30 leaders serve under the direct supervision of a Health Extension Worker. They are supposed to help educate and organize the 1–5 leaders and members for various activities.

Thus both 1–5 and 1–30 leaders, as unpaid volunteers, are supposed to take some of the burden of outreach off the shoulders of HEWs, who previously were tasked with leading all women in their catchment area towards a “healthy lifestyle” [21, 25, 26]. Women’s Development Army leaders ideally help during immunization campaigns, keep track of pregnancies and illnesses, and relay messages and data between households and HEWs. They are expected to hold weekly meetings with their members to discuss issues related to children’s health, hygiene, nutrition, antenatal care, birth, and so on. They receive no pay, and government policy is that they receive no incentives of any kind from donors, NGOs, or other partners.

## Methods

This paper reports primarily on survey data collected in 2015. The survey we conducted was part of a larger, mixed-methods study involving qualitative ethnographic work. The qualitative research, conducted from 2011 to 2016, included a document review, interviews, observations, and focus group discussions in six *kebeles* (local administrative units) within three districts (North Achefer, South Achefer, and Mecha) in West Gojjam zone, Amhara Regional State. Our qualitative work deeply informed our cross-sectional survey design, including our sampling strategy and the questions we asked of participants.

### Sampling

We surveyed 422 women in total, including 1–30 leaders, 1–5 leaders, and “members” (community members not selected as leaders) in the Women’s Development Army. For the survey, we selected four kebeles in which we had previously conducted qualitative research activities. The four kebeles were diverse in terms of distance to a paved road, accessibility, and level of activity/organization of the Women’s Development Army as reported by key informants.

To achieve a random sample in each kebele, we first asked Health Extension Workers to prepare for us their own lists of current 1–30 leaders within the WDA. The number of 1–30 leaders on their lists ranged from 28 to 56. We then used a random number generator to select 15 to 25 1-to-30 leaders from each list. With the help of HEWs and other local guides, one of the authors (RA) approached the randomly selected 1–30 leaders to complete surveys. At the end of each survey, she asked the 1–30 leader to name each of the 1–5 leaders under her supervision. Using a six-sided game die, she randomly selected one to two of the 1–5 leaders from the list. She then approached them and completed surveys. She then asked each surveyed 1–5 leader to name the women with whom she was expected to meet, and followed the same procedure for randomly selecting two to three of these 1–5 members for surveys. This procedure was followed until we reached a sample of *n* = 422, including 73 1–30 leaders, 142 1–5 leaders, and 207 1–5 members.

### Survey measures

We asked a variety of questions about respondents’ demographics, education, assets, and access to government services. Regarding assets, we asked whether or not respondents owned a series of eight agricultural assets (oxen, cows, chickens, sheep, donkeys, horses, economic trees, vegetable garden), and a series of eight other household assets (phone, radio, television, fridge, table, chair, bed, watch). We also used survey measures to evaluate respondents’ levels of food security, water security, stressful life events, social support, work burden, and psychological distress.

Household food insecurity refers to a situation in which individuals or households experience physically, socially, and/or economically restricted access to food of sufficient quantity and quality (including cultural preference) for a healthy life. We measured household food insecurity with a version of the Household Food Insecurity Access Scale (HFIAS) [27, 28]. The HFIAS has previously been validated and used by several research teams in rural and urban Ethiopia [19, 20, 29, 30].

Household water insecurity occurs when individuals or households experience restricted access to sufficient and safe water for their various needs, including economic, cultural, psychological, social, and spiritual needs [30–32]. Ethiopia is one of a small number of locations in which researchers have worked to develop a measure of household water insecurity. We measured water insecurity guided by methods developed in Ethiopia [30, 33].

We assessed the distribution of stressful life events by asking women whether or not they had experienced 16 different events, including violence and severe forms of deprivation or loss. We developed the list of events based on the results of focus group discussions with women that we conducted in 2013: participants were asked what kinds of difficult or challenging events are commonly experienced by women in their communities.

We assessed the distribution of common psychological distress symptoms (i.e. depression, anxiety, and somatoform) with a 29-item version of the WHO Self-Reporting Questionnaire, known as the SRQF [34]. The SRQF was adapted for Amharic-speaking populations, and incorporates eight items derived from Amharic idioms of distress (e.g. feeling that someone has cursed you; feeling that your heart is beating too fast). The SRQF has been tested for content, construct and criterion validity [35], and used in previous population research in Ethiopia [19, 36]. Participants were presented with ‘yes’ or ‘no’ response categories for each SRQF item/symptom. Affirmative responses were coded as 1 and negative responses as 0. The number of affirmative responses was summed to create a psychological distress symptom score (out of 29) for each individual.

Surveys of deprivation and forms of suffering are prone to response biases, including social desirability bias (for example, under-reporting deprivation because it is shameful) and other biases, for instance over-reporting deprivation because respondents think that perhaps doing so will get them resources. While avoiding such biases entirely is not possible, we attempted to construct and administer the survey in a way that would reduce such biases, both in the wording of the questions and the order they were asked (for example, putting more sensitive topics near the end of the survey).

### Analysis

This paper reports descriptive statistics (frequencies, means, and ranges) for our three sample strata: 1–30 leaders, 1–5 leaders, and 1–5 members. We pooled data from the four kebeles in which surveys were conducted. Data were entered by trained undergraduates, and then checked for errors and analyzed in SPSS v.23.

We report *p*-values associated with comparisons between 1-30 leaders and the rest of our sample (1–5 leaders and members combined). Initial analyses suggested that there were few differences between 1-5 leaders and members. Also, 1–30 leaders are of particular interest since they are tasked with greater community health worker responsibilities. For continuous variables, we used *t*-tests to compare 1–30 leaders to the rest of the sample. For categorical variables, we used Pearson Chi-square tests or Fisher’s exact tests. We also report Cronbach’s alpha as a measure of scale reliability for our measures of food insecurity, water insecurity, social support, and psychological distress. A Cronbach’ s alpha value of 0.70 or higher is commonly considered to indicate an acceptable level of scale reliability for health-related measures such as these.

## Results

In our interviews, many women told us that they engaged in 1–30 leadership work because they hoped it would lead to access to resources from the government. A few 1–30 leaders were able to get some government income through additional activities like attending trainings and providing adult education to others. Most 1–30 leaders, however, were unable to access government resources through 1–30 work. The survey results we report here advance understanding of these women’s social, economic, and psychological circumstances.

### Socio-economic and demographic indicators

Table 1 shows basic demographic and socio-economic status indicators. Taken in global context, the respondents in this study were deeply impoverished. Few respondents had access to basic amenities like electricity or mobile phones. The large majority of respondents had never been to school. The majority of respondents possessed some farmland, but as in most of highland Amhara, the size of most women’s farm plots is very small. A majority of respondents said they owned their house, as is common in rural Amhara (we did not collect information on joint- versus sole-ownership). Most houses are constructed by the owners, and consist of wooden poles, mud, and thatch or corrugated iron, and lack well-functioning latrines and other basic infrastructures. A majority of women participated in some non-farm income-generating activity. The two most common income-generating activities were selling home-distilled alcohol (*areke*) to wholesalers and selling homegrown vegetables at market.

**Table 1:** Socio-economic and demographic indicators among *n* = 422 Women’s Development Army leaders and members, rural Amhara, 2015

	1–30 Leaders (*n* = 73)	1–5 Leaders (*n* = 142)	1–5 Members (*n* = 207)	*P*-values^b^
Age in years, mean (range)	36.6 (18–55)	34.2 (18–60)	34.7 (18–65)	0.096
Married, %	61.6	80.3	82.6	0.000
Divorced or separated, %	21.9	7.7	7.7	0.000
Widowed, %	15.1	11.3	8.7	0.180
Any formal schooling, %	23.3	17.6	13	0.112
Schooling in years, mean (range)	1.2 (0–10)	0.9 (0–11)	0.7 (0–10)	0.223
People in household, mean (range)	4.8 (2–9)	5.4 (1–12)	5.0 (1–10)	0.124
Own oxen, %	61.6	78.9	79.2	0.001
Own donkeys, %	15.1	23.9	25.6	0.070
Own mobile phone, %	11	3.5	4.8	0.041^a^
Own house, %	95.9	99.3	96.1	0.444^a^
Have land to farm, %	84.9	90.1	84.5	0.668
Have electricity in your house, %	6.8	6.3	6.8	1.000^a^
Non-farm income-generating activity, %	74.0	65.5	61.8	0.082
Received micro-loans, %	53.4	45.8	38.6	0.063

^a^Fisher’s exact test

^b^All *p*-values in this paper are associated with comparisons between 1-30 leaders and the rest of our sample (1–5 leaders and members combined)

1–30 leaders were less likely to own oxen and more likely to own a mobile phone. 1–30 leaders were also less likely to be married and more likely to be divorced or separated. In our interviews, divorced women expressed their relief for the safety divorce afforded them, and expressed strong support for this important right, a point to which we return in the discussion.

### Work burden

**Fig. 1:**
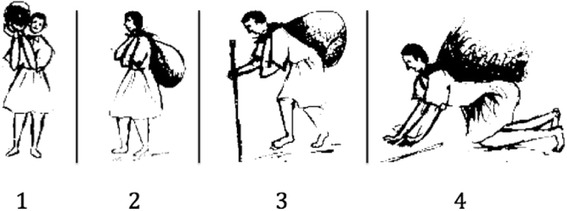
Pictorial scale used to quantify work burdens

We quantified women’s work burdens by asking respondents to rate their perceived work burden in the 5 domains listed in Table 2. Women ranked their burden for each item from 0 (no work) to 4 (heaviest burden), aided by a sketch of a woman physically burdened by a heavy pack, pictured in Fig. 1. Higher scores indicate higher self-reported workloads. We summed answers to the 5 questions to create an overall workload score that could range from 0 to 20. The composite workload score reveals that, on average, 1–30 leaders rated higher on our scale, mainly due to having a higher health/development-related work burden.

**Table 2:** Workloads among *n* = 422 Women’s Development Army leaders and members, rural Amhara, 2015

	1–30 Leaders (*n* = 73)	1–5 Leaders (*n* = 142)	1–5 Members (*n* = 207)	*P*-values
Farm work burden, mean (range)	2.9 (0–4)	3.1 (0–4)	2.8 (0–4)	0.925
Household chore burden, mean (range)	2.8 (1–4)	2.8 (1–4)	2.8 (1–4)	0.648
Childcare burden, mean (range)	1.4 (0–4)	1.9 (0–4)	1.6 (0–4)	0.091
Income-generating work burden, mean (range)	2.3 (0–4)	2.6 (0–4)	2.1 (0–4)	0.073
Health/development-related work, mean (range)	1.9 (0–4)	0.8 (0–4)	0.3 (0–3)	0.000
Composite workload score, mean (range)	11.6 (4–19)	10.7 (3–20)	9.4 (3–19)	0.000

### Food insecurity

The nine items in the Household Food Insecurity Access Scale (HFIAS) ask about increasingly severe experiences of household food insecurity in the previous 30 days. ‘No’ responses are coded as 0. ‘Yes’ responses to each item are followed up with a question about frequency of experience, with three possible responses: rarely (Amharic: *alfo alfo*, coded as 1), sometimes (*andand gize,* coded as 2), and often (*bizu gize*, coded as 3). Cronbach’s alpha for the food insecurity scale was 0.85. In Table 3, we report the percentage of ‘yes’ responses to each item, as well as an average food insecurity score calculated by summing an individual’s responses to each item [27, 28]. Food insecurity scores could range from 0 to 27. 1–30 leaders were slightly more likely than 1–5 leaders and members to answer yes to each item in the HFIAS; the difference was statistically significant for items 5 and 6. 1–30 leaders also had higher average food insecurity scores, but the difference was not statistically significant.

**Table 3:** Food Insecurity among *n* = 422 Women’s Development Army leaders and members, rural Amhara, 2015

HFIAS items	1–30 Leaders (*n* = 73)	1–5 Leaders (*n* = 142)	1–5 Members (*n* = 207)	*P*-values
1: Worried about having enough food, % yes	35.6	35.2	31.4	0.661
2: Not able to get preferred foods, % yes	38.4	32.4	27.1	0.124
3: Ate just a few kinds of food, % yes	47.9	42.3	42	0.361
4: Ate unwanted/undesirable food, % yes	12.3	9.2	7.7	0.275
5: Reduced amount of food eaten, % yes	27.4	22.5	13.5	0.043
6: Ate fewer meals/times in a day, % yes	23.3	14.1	12.1	0.023
7: No food at all in the house, % yes	5.5	2.1	1.9	0.104^a^
8: Went to sleep hungry/without eating, % yes	8.2	4.2	4.8	0.242^a^
9: Went a whole day without eating, % yes	5.5	2.1	2.4	0.136^a^
Food Insecurity score, mean (range)	3.0 (0–22)	2.6 (0–19)	2.2 (0–20)	0.149

^a^Fisher’s exact test

### Water insecurity

Table 4 lists the 22 water insecurity items. ‘Yes’ responses were coded as 1 and ‘no’ responses as 0. We report the percentage of ‘yes’ responses to each item, as well as a water insecurity score calculated by summing an individual’s responses. Cronbach’s alpha for the water insecurity scale was 0.94. 1–30 leaders were slightly more likely than 1–5 leaders and members to answer yes to some water insecurity items, and had higher average water insecurity scores overall; however, none of these differences was statistically significant.

**Table 4:** Water Insecurity among *n* = 422 Women’s Development Army leaders and members, rural Amhara, 2015

	1–30 Leaders (*n* = 73)	1–5 Leaders (*n* = 142)	1–5 Members (*n* = 207)	*P*-values
Drank water that might not be safe	43.8	40.8	38.6	0.496
Did not collect because it takes too long to queue	41.1	38	33.3	0.344
Slept very few hours due to early morning water collection	35.6	32.4	29	0.379
Worried about not having enough water for all household needs	34.2	29.6	34.8	0.794
Did not complete chores/work due to water collection	30.1	28.2	24.2	0.444
Borrowed water from a neighbor due to lack at home	28.8	29.6	24.2	0.673
Reduced water for drinking	27.4	28.9	26.6	0.985
Did not collect water because source was too far	26	23.2	27.5	0.996
Reduced water for cooking	21.9	19	18.8	0.555
Reduced water for bathing	21.9	16.9	21.3	0.636
Reduced water for making coffee/tella	19.2	16.2	17.4	0.641
Reduced water for washing clothes	19.2	16.9	20.3	0.958
Did not cook a desirable food due to lack of water	17.8	14.1	15.9	0.575
Did not collect because it was too dangerous/risky to go	17.8	17.6	11.1	0.370
Reduced water for washing utensils	13.7	14.1	19.8	0.432
Did not collect because there was not enough water at source	13.7	15.5	16.4	0.616
Reduced water for washing face, hands, and/or feet	12.3	11.3	15.5	0.746
Reduced water for cleaning house	11	10.6	13	0.796
Collected water from a dirty or undesirable source	11	9.9	11.6	0.986
Went to sleep thirsty	11	11.3	11.6	0.902
Quarreled with a neighbor or other person over water	5.5	4.2	4.3	0.755^a^
Went whole day without drinking water	5.5	2.8	5.8	0.762^a^
Water Insecurity score, mean (range)	4.7 (0–21)	4.3 (0–20)	4.4 (0–22)	0.661

^a^Fisher’s exact test

### Stressful life events

Table 5 reports information on stressful life events. Respondents were asked whether they had experienced each event in the last year. We created a stressful life events score by summing an individual’s responses. Women in our sample commonly experienced debt and time periods in which they had no money at all. 1–30 leaders were slightly more likely to have been in debt and to have been hurt by their husband or another man (although these differences did not reach significance). 1–30 leaders were significantly more likely to report having been the subject of local gossip (*p* = 0.019), and experienced a significantly greater number of stressful life events (*p* = 0.007).

**Table 5:** Stressful Life Events among *n* = 422 Women’s Development Army leaders and members, rural Amhara, 2015

	1–30 Leaders (*n* = 73)	1–5 Leaders (*n* = 142)	1–5 Members (*n* = 207)	*P*-values
Household debt, %	53.4	48.6	38.6	0.093
Serious illness, %	42.5	35.9	38.2	0.404
No money at all in past year, %	42.5	45.8	35.7	0.676
Death of close family, spouse, or friend, %	28.8	24.6	22.7	0.340
Subject to local gossip, %	28.8	17.6	16.4	0.019
Major crop loss or damage, %	24.7	24.6	18.8	0.516
No land at all, %	9.6	7.7	10.1	0.910
Unusually difficult pregnancy or birth, %	9.6	6.3	6.3	0.312
Abuse/serious problem from a man, %	6.8	4.2	2.9	0.190
Observed fight/accident with severe injury, %	6.8	1.4	3.9	0.153
Unwanted pregnancy, %	5.5	2.8	3.4	0.306
Unusually difficult/troublesome child, %	5.5	4.9	4.3	0.762
Serious injury, %	4.1	1.4	0.5	0.067
Unable to access health care when very sick, %	4.1	2.8	6.3	1.000
Land dispute or taken away, %	2.7	2.1	2.4	0.686
Person or house robbed, %	0	2.1	1	0.593
Number of stressful life events, mean (range)	2.7 (0–7)	2.3 (0–7)	2.1 (0–8)	0.007

### Psychological and social wellbeing

Table 6 reports participants’ level of perceived social support. We summed responses to seven questions that asked participants if it would be “very easy” (=1), “easy” (=2), “difficult” (=3), or “very difficult” (=4) to: get someone to watch their children; borrow a small amount of salt or coffee; get help with a big task such as building, farming or repairing; borrow 25 kg of flour; borrow money to buy medicine for their children; borrow or get 10 birr (~ 0.5 USD); and borrow or get 50 birr (~ 2 USD). Scores could range from 7 to 28, with higher scores indicating lower perceived social support. Cronbach’s alpha for this measure of social support was 0.86. The majority of women in each sample category reported that it was easy or very easy to get the kind of support indexed in each item, with the exception of borrowing 25 kg of flour (most said this was difficult or very difficult; data not shown). 1–30 leaders on average reported slightly lower levels of social support (*p* = 0.048).

**Table 6:** Psychosocial wellbeing among *n* = 422 Women’s Development Army leaders and members, rural Amhara, 2015

	1–30 Leaders (*n* = 73)	1–5 Leaders (*n* = 142)	1–5 Members (*n* = 207)	*P*-values
Social Support Score, mean (range)	15.8 (7–28)	14.4 (7–27)	14.1 (7–28)	0.048
Psychological Distress Symptoms, mean (range)	6.3 (0–24)	5.3 (0–22)	4.7 (0–24)	0.022
8 or more psychological distress symptoms, %	37	26	22	0.017

Table 6 also reports psychological distress symptom loads as measured by the SQRF. Cronbach’s alpha for this measure of psychological distress was 0.84. 1–30 leaders reported an average of six symptoms, significantly more than the rest of the sample (*p* = 0.022). Though the SRQF is not a diagnostic tool, a cutoff of 7/8 was determined by Zilber and colleagues to be optimal for screening common mental disorder (CMD) cases from urban Ethiopian populations [35]. We use this cutoff to report the percentage of respondents who would likely be diagnosed with a CMD. 1–30 leaders were significantly more likely to exceed the cutoff of eight or more psychological distress symptoms (*p* = 0.017).

The most commonly reported symptoms across all sample categories were frequent headaches (57%), feeling that the heart is beating too fast (37%), easily getting angry at others (36%), uncomfortable feelings in the stomach (33%), and feeling easily tired (30%).

### Desires for government intervention

Finally, Table 7 summarizes women’s responses regarding the primary thing that they want the Ethiopian government to do (or do better) to improve their lives. The most common response was “give money” across all respondent categories, which in general refers to money extended in the form of micro-loans. Many women in our study area have received micro-loans through government programs. Women with whom we spoke clarified that while government subsidized micro-credit programs have been helpful in some cases, they worry about their ability to pay interest and the risk of falling into debt. 80% of our survey respondents also agreed with the statement that Women’s Development Army leaders should be paid for their work (data not shown).

**Table 7:** “What the government should provide to improve women’s lives,” according to *n* = 422 Women’s Development Army leaders and members, rural Amhara, 2015

	1–30 Leaders (*n* = 73)	1–5 Leaders (*n* = 142)	1–5 Members (*n* = 207)
Give money, %	30.1	36.6	35.3
Other (education, farming support), %	24.7	16.2	14
Give jobs, %	15.1	6.3	6.8
Give land, %	9.6	21.1	25.1
Improve health care, %	8.2	7	8.2
Improve water access, %	6.8	8.5	6.8
Nothing, %	2.7	2.1	3.4
Give food aid, %	1.4	0.7	0
Don’t know, %	1.4	1.4	0.5

## Discussion

Our survey shows that WDA leaders in rural Amhara are not very different than rural women who are not becoming volunteer CHWs: large proportions experience insecure access to food and water, own few assets, have very low levels of schooling, have heavy workloads combining work in several domains, and are in debt. Like the populations they serve, these volunteer CHWs often experience scarcity.

Our survey also revealed some differences between 1-30 leaders and other women that warrant attention. 1–30 leaders were more likely to experience some aspects of food insecurity and reported greater levels of psychological distress and more stressful life events. They also reported slightly less social support than other women. Taken together, these findings illustrate that 1–30 leaders are a population living in precarity, in some realms experiencing even more hardship than other women in our sample.

These findings add to the epidemiological literature on food and water insecurity and psychosocial distress in Ethiopia and globally. Using the HFIAS in a mix of rural and urban households in Butajera district in south-central Ethiopia, Gebreyesus and colleagues (2015) found mean food insecurity scores of 6.1 to 6.3 [29]. Stevenson and colleagues (2016) found mean HFIAS scores of 3.1 to 5.6, and mean water insecurity scores of 1.2 to 3.1 among rural households in South Wello, another zone located in Amhara state [37]. That study examined water insecurity before and after construction of new protected wells and springs, sponsored by government-NGO partnerships. Implementation of new water access points was followed by a significant decline in household water insecurity in the intervention sites. In another study carried out across multiple regions of Ethiopia, Hadley and Freeman (2016) found a significant decline in household water insecurity scores subsequent to water, sanitation, and hygiene interventions carried out through NGO-government partnership [33].

A few studies have measured food insecurity and/or psychological distress specifically among CHWs in Ethiopia. Dynes and colleagues (2014) used a version of the HFIAS to measure food insecurity among Health Extension Workers, Community Health Development Agents, and traditional birth attendants in seven kebeles in West Gojjam, Amhara state. They collected their data prior to the re-organization of the ranks they called Community Health Development Agents into the WDA ranks that we studied. Mean HFIAS scores in that study were 1.0 for Health Extension Workers, 2.0 for unpaid Community Health Development Agents, and 6.0 for traditional birth attendants [20]. Maes and colleagues used the SRQF and reported high rates of psychological distress symptoms among unpaid CHWs in Addis Ababa [18, 19]. CHWs in that study also reported high levels of food insecurity as assessed with the HFIAS. Importantly, all of the studies that measured both psychological distress and water and/or food insecurity found that insecure access to food and water are correlated with psychological distress. These studies further suggest that food and water insecurity generate psychological distress by introducing worry, uncertainty and shame, as well as hunger and thirst into peoples’ lives, and by eroding norms of reciprocity and sociality that depend on food and water [30, 38–40].

Thus the women in our sample share problems like food and water insecurity and psychological distress with many other populations across Ethiopia. Variation in chronicity and severity of household food and water insecurity is also apparent, driven by several ecological factors operating from local to global levels. These include seasonality, availability and access to infrastructure, price fluctuations, livelihood insecurity, lack of employment and income, and variation in social status. Anthropologists Wutich and Brewis (2014) propose that entitlement theory should guide understanding of the determinants of water and food insecurity [41]. This theoretical approach intersects with political ecology and critical medical anthropology, which seek to understand how institutional, social, and political-economic trends interact with physical ecology and climate to produce resource scarcity in specific populations. While physical ecology and population size play roles in shaping food and water security, entitlement failures involving governance and/or market failures are more important. Further, while failures of private markets often play a greater role in generating food insecurity, failures of states and public-private partnerships—in planning, expenditure, infrastructure provision, and environmental regulation—tend to play a bigger role in generating water insecurity, in part because water systems are harder to privatize compared to food systems [40]. The studies from Ethiopia cited above and others appear to support this general understanding, though further research is needed to show how specific variables and processes lead to food and water insecurity in specific ecologies [42, 43].

State policy and rhetoric that 1–30 leaders are “model women” who come from “model households” would suggest that they enjoy better socioeconomic status in comparison to other rural women. Our work suggests a more complicated but understandable reality. The qualitative data suggest that, when it comes to recruiting 1–30 leaders, local health officials do not necessarily have the luxury of recruiting so-called “model women,” and instead in many instances struggle to persuade *any* women to take on the unpaid role. In a context in which the government has resisted providing any incentives at all for WDA leaders [39], these CHW jobs are filled by vulnerable women who hope the work will ultimately bring some benefit.

Our survey findings further revealed that 1–30 leaders were less likely to be married and more likely to be divorced or separated. Based on our qualitative research, we suspect that 1–30 leaders are more likely to be divorced or separated because women who are divorced or separated tend to lead socio-economically precarious lives while also enjoying the freedom of separation from abusive and/or overbearing spouses. These women may be more likely to take on the 1–30 role because they hope that it will bring them some socioeconomic benefit, and because they are free to make the choice for themselves. Further research is needed to test these hypotheses and develop our understanding of CHW recruitment in rural Ethiopia.

According to the 2016 Ethiopia Demographic and Health Survey (DHS), Ethiopian women who are divorced or separated are more likely to have experienced violence, including sexual and spousal violence, in their life [44]. Across Ethiopia, divorced, separated and widowed women’s households also have less land and livestock, and less social support, than married women’s households [45]. This may partially explain why 1–30 leaders in our study were more distressed and deprived than their neighbors. The Ethiopian government’s efforts to make it easier for women to get divorces from abusive husbands and to protect their property rights after divorce are highly valued by many women in Ethiopia, including respondents in our study [45]. Protecting these freedoms has certainly led to improved wellbeing for many Ethiopian women, including women in our sample. Our findings underline the importance of ensuring that Ethiopian WDA leaders, regardless of marital status, also have secure livelihoods and access to basic resources including food and water.

Our findings are particularly useful in evaluating Ethiopia’s primary health care system policies. Our qualitative work and other studies show that modestly paid Health Extension Workers are, although certainly far from wealthy, slightly better off than their neighbors [20]. They are better off in part because they benefit from their incomes as paid public employees. These positions, in turn, were extended to them because they had completed at least 10 years of schooling, according to official Ministry of Health policy. As noted in the introduction, the previous Minister of Health of Ethiopia (now Director General of the World Health Organization), Dr. Tedros Adhanom, claimed that the success and sustainability of the Health Extension Program depended on providing a regular salary to health extension workers and thereby “moving away from volunteerism” [23, 46]. This policy and rhetoric are contradicted by the later policy decision to shift tasks from paid Health Extension Workers to unpaid Women’s Development Army leaders. With the Women’s Development Army, the Ethiopian Health Extension Program has actually deepened and expanded an already massive reliance on unpaid women’s labor. The political economic constraints, competing priorities, and logic behind this contradiction are complex [47]. An evaluation of this policy contradiction in terms of its current or future impacts on population health and primary health care access are beyond the scope of this article. Our intention is to show that the decision to shift tasks from paid HEWs to Women’s Development Army leaders has resulted in the recruitment of a massive amount of unpaid labor by the state, provided by women who already shoulder heavy work burdens and who face alarming and unacceptable levels of food and water insecurity and psychological distress.

There is clearly a need for much higher funding for primary health care in poor countries like Ethiopia, including funding for paying a larger number of effective community health workers [48, 49]. With appropriate funding levels, Ethiopia’s Ministry of Health could create more paid HEW positions and/or paid WDA leader positions for women who have fewer years of schooling. Our results should encourage Ethiopian policy makers, international donors, and rural Ethiopian women themselves to openly discuss these questions of funding, primary health care labor remuneration, and other related policies.

## Conclusions

As long as CHWs remain unpaid members of the impoverished populations that global health programs aim to serve, they will stay impoverished. Given that health is deeply tied to socioeconomic status, paying CHWs for their labor is a straightforward way not only to motivate them in their work, but also to more deeply improve health outcomes within their own households and communities. Beyond addressing food insecurity and other economic challenges, wages could interact in synergistic ways with other efforts to genuinely empower women.

## Abbreviations

CHW: Community Health Worker
CMD: Common Mental Disorder
DHS: Demographic and Health Survey
HEW: Health Extension Worker
HFIAS: Household Food Insecurity Access Scale
NGO: Non-Governmental Organization
SRQF: Falk Self-Reporting Questionnaire for Amharic Speaking Populations
USD: United States Dollar
WDA: Women’s Development Army
WHO: World Health Organization

## References

[1] WHO (1989). Strengthening the performance of community health workers in primary health care.

[2] Wiggins N, Kaan S, Rios-Campos T, Gaonkar R, Morgan ER, Robinson J (2013). Preparing community health Workers for Their Role as agents of social change: experience of the community capacitation center. Journal of Community Practice.

[3] Perry HB, Zulliger R, Rogers MM (2014). Community health Workers in low-, middle-, and high-income countries: an overview of their history, recent evolution, and current effectiveness. Annu Rev Publ Health.

[4] Bhattacharyya K, LeBan K, Winch P, Tien M (2001). Community health worker incentives and disincentives: how they affect motivation, retention, and sustainability.

[5] Alam K, Oliveras E (2014). Retention of female volunteer community health workers in Dhaka urban slums: a prospective cohort study. Hum Resour Health.

[6] Ashraf N, Bandiera O, Jack BK (2014). No margin, no mission? A field experiment on incentives for public service delivery. J Public Econ.

[7] McCord GC, Liu A, Singh P (2013). Deployment of community health workers across rural sub-Saharan Africa: financial considerations and operational assumptions. Bull World Health Organ.

[8] Patel AR, Nowalk MP (2010). Expanding immunization coverage in rural India: a review of evidence for the role of community health workers. Vaccine.

[9] Kok MC, Broerse JEW, Theobald S, Ormel H, Dieleman M, Taegtmeyer M (2017). Performance of community health workers: situating their intermediary position within complex adaptive health systems. Hum Resour Health.

[10] Closser S, Rosenthal A, Justice J, Maes K, Sultan M, Banerji S (2017). Per diems in polio eradication: perspectives from community health workers and officials. Am J Public Health.

[11] Nyirenda L, Flikke R (2013). Frontline vaccinators and immunisation coverage in Malawi. Forum for Development Studies.

[12] Swartz A, Colvin CJ (2014). It’s in our veins’: caring natures and material motivations of community health workers in contexts of economic marginalisation. Critical Public Health.

[13] Mumtaz Z, Salway S, Waseem M, Umer N (2003). Gender-based barriers to primary health care provision in Pakistan: the experience of female providers. Health Policy Plan.

[14] Mumtaz Z, Salway S, Nykiforuk C, Bhatti A, Ataullahjan A, Ayyalasomayajula B (2013). The role of social geography on lady health workers’ mobility and effectiveness in Pakistan. Soc Sci Med.

[15] Ramirez-Valles J (1998). Promoting health, promoting women: the construction of female and professional identities in the discourse of community health workers. Soc Sci Med.

[16] Justice J (1984). Can socio-cultural information improve health planning? A case study of Nepal’s assistant nurse-midwife. Soc Sci Med.

[17] Maes K, Kalofonos I (2013). Becoming and remaining community health workers: perspectives from Ethiopia and Mozambique. Soc Sci Med.

[18] Maes KC, Hadley C, Tesfaye F, Shifferaw S, Tesfaye YA (2009). Food insecurity among volunteer AIDS caregivers in Addis Ababa, Ethiopia was highly prevalent but buffered from the 2008 food crisis. J Nutr.

[19] Maes KC, Hadley C, Tesfaye F, Shifferaw S (2010). Food insecurity and mental health: surprising trends among community health volunteers in Addis Ababa, Ethiopia during the 2008 food crisis. Soc Sci Med.

[20] Dynes MM, Stephenson R, Hadley C, Sibley LM (2014). Factors shaping interactions among community health workers in rural Ethiopia: rethinking workplace trust and teamwork. J Midwifery Womens Health.

[21] Teklehaimanot HD, Teklehaimanot A (2013). Human resource development for a community-based health extension program: a case study from Ethiopia. Hum Resour Health [Internet].

[22] Central Statistical Agency, ICF International (2011). Ethiopia Demographic and Health Survey.

[23] WHO (2009). Ethiopia extends health to its people: An interview with Dr. Tedros A Ghebreyesus. Bull World Health Organ.

[24] WHO. Task Shifting: Rational Redistribution of Tasks Among Health Workforce Teams - Global Recommendations and Guidelines [Internet]. Geneva: World Health Organization; 2008. Available from: Available from: http://data.unaids.org/pub/Manual/2007/ttr_taskshifting_en.pdf [accessed on 9 Nov 2009].

[25] FMOH (2011). HSDP IV annual performance report EFY 2003 (2010/2011).

[26] CNHDE. Evaluation of the Health Extension Program Implementation Process and Effect on Health Outcomes Part III: Model-Family and vCHP Survey. Center for National Health Development in Ethiopia, Columbia University, in collaboration with the FMOH of Ethiopia, UNICEF, and WHO; 2011.

[27] Swindale A, Bilinsky P (2006). Development of a universally applicable household food insecurity measurement tool: process, current status, and outstanding issues. J Nutr.

[28] Coates J, Swindale A, Bilinsky P (2007). Household food insecurity access scale (HFIAS) for measurement of food access: indicator guide.

[29] Gebreyesus SH, Lunde T, Mariam DH, Woldehanna T, Lindtjørn B (2015). Is the adapted household food insecurity access scale (HFIAS) developed internationally to measure food insecurity valid in urban and rural households of Ethiopia?. BMC Nutrition.

[30] Stevenson EGJ, Greene LE, Maes KC, Ambelu A, Tesfaye YA, Rheingans R (2012). Water insecurity in 3 dimensions: an anthropological perspective on water and women’s psychosocial distress in Ethiopia. Soc Sci Med.

[31] Hadley C, Wutich A (2009). Experience-based measures of food and water security: biocultural approaches to grounded measures of insecurity. Hum Organ.

[32] Wutich A, Ragsdale K (2008). Water insecurity and emotional distress: coping with supply, access, and seasonal variability of water in a Bolivian squatter settlement. Soc Sci Med.

[33] Hadley C, Freeman MC (2016). Assessing reliability, change after intervention, and performance of a water insecurity scale in rural Ethiopia. Food Security.

[34] World Health Organization (1994). A user’s guide to the self reporting questionnaire (SRQ).

[35] Zilber N, Youngmann R, Workneh F, Giel R (2004). Development of a culturally-sensitive psychiatric screening instrument for Ethiopian populations.

[36] Hanlon C, Medhin G, Alem A, Araya M, Abdulahi A, Hughes M (2008). Detecting perinatal common mental disorders in Ethiopia: validation of the self-reporting questionnaire and Edinburgh postnatal depression scale. J Affect Disord.

[37] Stevenson EGJ, Ambelu A, Caruso BA, Tesfaye Y, Freeman MC (2016). Community water improvement, household water insecurity, and Women’s psychological distress: an intervention and control study in Ethiopia. PLoS One.

[38] Amare Y (2010). Urban food insecurity and coping mechanisms: a case study of Lideta sub-city in Addis Ababa.

[39] Hadley C, Stevenson EGJ, Tadesse Y, Belachew T (2012). Rapidly rising food prices and the experience of food insecurity in urban Ethiopia: impacts on health and well-being. Soc Sci Med.

[40] Maes KC, Shifferaw S, Hadley C, Tesfaye F (2011). Volunteer home-based HIV/AIDS care and food crisis in Addis Ababa, Ethiopia: sustainability in the face of chronic food insecurity. Health Policy & Planning.

[41] Wutich A, Brewis A (2014). Food, water, and scarcity: toward a broader anthropology of resource insecurity. Curr Anthropol.

[42] Jepson W, Budds J, Eichelberger L, Harris L, Norman E, O’Reilly K (2017). Advancing human capabilities for water security: a relational approach. Water Security.

[43] Wutich A, Budds J, Eichelberger L, Geere J, Harris LM, Horney JA (2017). Advancing methods for research on household water insecurity: studying entitlements and capabilities, socio-cultural dynamics, and political processes, institutions and governance. Water Security..

[44] Central Statistical Agency (CSA) [Ethiopia] and ICF (2016). Ethiopia Demographic and Health Survey.

[45] Kumar N, Quisumbing AR (2015). Policy Reform toward gender equality in Ethiopia: little by little the egg begins to walk. World Dev.

[46] FMOH (2010). Ethiopia’s fourth National Health Accounts, 2007/2008.

[47] Maes K, Closser S, Vorel E, Tesfaye Y (2015). A Women’s development Army: narratives of community health worker investment and empowerment in rural Ethiopia. St Comp Int Dev.

[48] Dahn B, Woldemariam AT, Perry H, Maeda A, von Glahn D, Panjabi R (2015). Strengthening Primary Health Care through Community Health Workers: Investment Case and Financing Recommendations.

[49] Ballard M, Schwarz, Ryan, Johnson, Ari, Church, Shaun, Palazuelos, Daniel, McCormick, Lisha, et al. Practitioner Expertise to Optimize Community Health Systems: Harnessing Operational Insight [Internet]. Sall Family Foundation; 2017. Report No.: DOI: 10.13140/RG.2.2.35507.94247. Available from: www.chwimpact.org

